# Muscle and joint mechanics during maximum force biting following total temporomandibular joint replacement surgery

**DOI:** 10.1007/s10237-023-01807-1

**Published:** 2024-03-19

**Authors:** Sarah C. Woodford, Dale L. Robinson, Jaafar Abduo, Peter V. S. Lee, David C. Ackland

**Affiliations:** 1 Department of Biomedical Engineering, University of Melbourne, Parkville, VIC 3010 Australia; 2 Melbourne Dental School, University of Melbourne, Parkville, VIC 3010 Australia

**Keywords:** Temporomandibular joint, Biomechanical model, Finite element analysis, Musculoskeletal model, Motion analysis, Chewing

## Abstract

Total temporomandibular joint replacement (TMJR) surgery is the established treatment for severe temporomandibular joint disorders. While TMJR surgery is known to increase mouth-opening capacity, reduce pain and improve quality of life, little is known about post-surgical jaw function during activities of daily living such as biting and chewing. The aim of this study was to use subject-specific 3D bite force measurements to evaluate the magnitude and direction of joint loading in unilateral total TMJR patients and compare these data to those in healthy control subjects. An optoelectronic tracking system was used to measure jaw kinematics while biting a rubber sample for 5 unilateral total TMJR patients and 8 controls. Finite element simulations driven by the measured kinematics were employed to calculate the resultant bite force generated when compressing the rubber between teeth during biting tasks. Subject-specific musculoskeletal models were subsequently used to calculate muscle and TMJ loading. Unilateral total TMJR patients generated a bite force of 249.6 ± 24.4 N and 164.2 ± 62.3 N when biting on the contralateral and ipsilateral molars, respectively. In contrast, controls generated a bite force of 317.1 ± 206.6 N. Unilateral total TMJR patients biting on the contralateral molars had a significantly higher lateral TMJ force direction (median difference: 63.6°, *p* = 0.028) and a significantly lower ratio of working TMJ force to bite force (median difference: 0.17, *p* = 0.049) than controls. Results of this study may guide TMJ prosthesis design and evaluation of dental implants.

**Supplementary Information:**

The online version contains supplementary material available at 10.1007/s10237-023-01807-1.

## Introduction

The temporomandibular joint (TMJ) is a bilateral synovial joint connecting the mandible to the skull which comprises the mandibular condyle, glenoid fossa of the temporal bone, and the articular cartilage and disc. Its function, which is to facilitate biting, chewing, swallowing and speech, is considered essential for maintaining quality of life. Painful disorders of the TMJ are common, with 36% of the population showing clinical signs of a TMJ disorder, including TMJ muscle and joint pain and tenderness, and limitations in mandibular function (Progiante et al. 2015). Total temporomandibular joint replacement (TMJR) surgery is the accepted approach for TMJ disorders such as degenerative joint disease, ankylosis and internal disc derangement when the condition cannot be managed by conservative treatment. Clinical studies demonstrate that TMJR surgery reduces pain, increases mouth-opening capacity, reduces dietary restrictions and improves patient quality of life (Dimitroulis et al. 2018; Zou et al. 2018; Woodford et al. 2023); however, unilateral TMJR patients often experience post-surgical degradation of their native contralateral joint, with up to 40% of patients requiring bilateral TMJR surgery within two years (Franco et al. 1997). While thought to be broadly related to joint loading (Perez et al. 2016; Linsen et al. 2021b), there is currently no consensus on mechanical factors associated with contralateral joint disease post-TMJR.

Bite force and jaw muscle and TMJ forces are important determinates of implant and joint functional performance following TMJR; however, they remain poorly understood post-operatively. Bite force has been measured using uniaxial transducers (Röhrle et al. 2018), with maximum voluntary bite force measurements for total TMJR patients ranging from 177 to 201 N (Linsen et al. 2021b; Speksnijder et al. 2022), substantially lower than that of healthy individuals. This functional decrease has been attributed to muscle atrophy and degeneration, lower occlusal contact area and psychological factors (Linsen et al. 2013). A limitation in these measurements is that data were recorded using a uniaxial transducer, which does not record lateral shear forces at the occlusal surface. Shear forces on dental structures are clinically relevant following unilateral TMJR due to the muscle force imbalance resulting from intraoperative severing of the lateral pterygoid muscle, or functional asymmetries such as reverse-sequence chewing patterns (Lewin & Ramadori 1985). A study of dynamic bite force in healthy subjects using a novel kinematics-driven simulation of mastication showed that shear forces can be as large as 42 N and 63 N on the central incisors and second molars, respectively (Woodford et al. 2021). These shear forces generate high stresses around the supporting bone of dental restorations (Pellizzer et al. 2011) and can be detrimental to the long-term integrity of dental implants (Forero et al. 2019). At present, however, 3D bite forces in TMJR patients have not been measured to date.

Resultant TMJ force is generated by contraction of the masticatory muscles, including the masseter and temporalis; however, no technology is available to quantify joint force in vivo. As a consequence, bite force is typically decomposed into both muscle and joint forces using computational modelling (Ackland et al. 2015, 2017, 2018; Abdi et al. 2020; Guo et al. 2022). In the native jaw, a subject-specific musculoskeletal model of the masticatory system was used to show that the maximum joint force is approximately 76% of the maximum voluntary bite force measured at the premolars (de Zee et al. 2007). This ratio is likely to be considerably more variable in TMJR patients, since the lateral pterygoid muscle is severed intraoperatively, and the masseter detached to accommodate the ramal component of the prosthesis (Linsen et al. 2020). Mathematical modelling of unilateral total TMJR subjects has demonstrated large prosthetic TMJ loading (van Loon et al. 1998), increased lateral joint loads (Ackland et al. 2017) and higher native contralateral joint stresses compared to healthy controls (Bekcioglu et al. 2017). However, these mathematical models employ bite and muscle forces derived from stomatognathically healthy control subjects, and these results are not applicable to the TMJR population.

The aim of this study was to use subject-specific 3D measurements of bite force to evaluate the magnitude and direction of joint reaction forces and the ratio of TMJ force to bite force in unilateral total TMJR patients and healthy controls. We hypothesised that joint reaction forces would have a significantly higher lateral component in unilateral total TMJR patients than healthy controls, and that unilateral TMJR patients would have a significantly lower ratio of TMJ force to bite force than healthy controls. The data produced in this study may help guide the design and evaluation of TMJ prosthesis and the prescription of physiotherapy following unilateral total TMJR surgery.

## Materials and methods

### Subject recruitment and imaging

Five unilateral total TMJR patients (mean age: 55.9 ± 6.4 yrs) and eight healthy control subjects (mean age: 46.0 ± 4.3 yrs) were recruited for testing. Total TMJR patients were fitted with the ArthroJaw TMJR system (Maxoniq, Melbourne); testing took place 12 to 15 months post-operatively (Table 1). Participants recruited were women who were aged forty years or over at the time of testing and had functional dentition. Participants were excluded if they experienced ongoing dental pain, were undergoing orthodontic treatment or were pregnant or breastfeeding. Control subjects with any prior occurrences of TMJ disorders were not included. Ethics approval for this study was provided by the institutional Human Ethics Advisory Group (HREC 1853328.1), and subjects provided written consent.

**Table 1:** Participant details, including age at time of data collection, joint replacement side, post-operative measurement time and mandible size as defined in Fig. 1d

	Age (years)	Joint replacement side	Measurement time post-op (years)	Mandible size	
				Mandibular length (mm)	Mandibular width (mm)
Control	48.5			85.3	92.3
	44.5			83.4	99.5
	42.9			76.2	96.9
	55.5			89.3	103.0
	42.2			89.8	105.9
	45.0			87.0	94.9
	44.1			95.5	108.3
	45.3			84.5	96.1
TMJR	59.1	Right	1.1	78.6	106.0
	55.7	Right	1.1	76.6	98.2
	64.9	Left	1.3	82.4	100.7
	51.4	Right	1.0	84.6	99.1
	48.6	Right	1.1	85.6	98.4

Each participant had a cone-beam computed tomography (CBCT) scan taken of their mandible, maxilla and bilateral TMJ’s, with a voxel size of 0.42 mm and a resolution of 0.5 mm (Aquilion One, Canon Medical Systems, USA). Participants were instructed to lie in a supine position with the mandible in the maximum occlusion position for the duration of the scan. Threshold-based segmentation was used to reconstruct the mandible and maxilla of each participant using commercial software (mimics 21.0 and 3-matic 13.0, Materialise, Belgium).

**Fig. 1:**
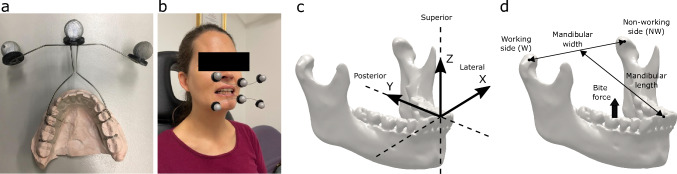
Custom-made motion tracking plates (**a**), participant with maxillary and mandibular tracking plates in place (**b**), anatomical coordinate system with X directed laterally (left), Y directed posteriorly and Z directed superiorly (**c**) and definitions of mandibular naming convention (**d**). Visible on the motion tracking plate are three retroreflective markers (top of image), clear lingual base plate, metal clasps around the second premolars and molars and wire extending extraorally connecting the clasps to the retroreflective markers. The working TMJ is defined as the TMJ on the same side as the bite; the non-working TMJ is defined as the TMJ on the opposite side to the bite

### Jaw motion experiments

Jaw motion experiments have been described previously (Woodford et al. 2023). Briefly, pairs of custom lightweight motion tracking plates were constructed to rigidly connect a triad of spherical retroreflective markers to each participant’s dental arches. Dental stone casts were manufactured from impressions taken of the mandibular and maxillary teeth, upon which the plates were developed (Fig. 1a). The plates consist of a polymethylmethacrylate base plate fixed to the second premolars and molars with stainless-steel wire clasps. A laser-welded wire attached to the base plate extended extraorally and was used to support three retroreflective markers (Fig. 1b). The plates were designed to facilitate complete occlusion of the teeth and unobstructed closure of the lips. Each motion tracking plate was scanned using a handheld laser scanner and a 3D surface model of the retroreflective markers and dental anatomy produced.

Pairs of custom motion tracking plates were fitted to the dental arches of each participant (Fig. 1b). Participants then supported a 20 mm × 20 mm × 6 mm soft silicone rubber sample between their teeth and were instructed to bite and chew on the sample until they felt familiar with the testing procedure. Rubber samples were centred between either the left or right first molars and aligned parallel to the mesiodistal axis. Each sample was in contact with 2–3 teeth per side, depending on the subject dentition. A photograph was taken of the rubber sample and surrounding teeth to record the rubbers position in relation to the dental arch (iPhone 12, Apple, California, USA). Each participant was then instructed to maximally compress the rubber between the selected teeth for three seconds, while the trajectories of the six retroreflective markers were captured using a 4-camera optoelectronic tracking system with a sampling frequency of 100 Hz and static resolution of 0.06 mm (Vero, Vicon, Oxford Metrics, UK). All motion capture data were filtered using a low-pass filter with a cut-off frequency of 10 Hz. This motion tracking system has a precision of 0.13 mm (see Supplementary Material). The testing protocol was repeated twice on the left and right molars in random order with rest breaks offered between each movement.

To evaluate the position and orientation of the mandibular and maxillary dental arches, 3D models of the mandible and maxilla of each participant were aligned with the optoelectronic tracking data. To accomplish this, the 3D models of each participant’s dental stone casts and motion tracking plates were mapped to the dental geometry from the CT scan with a global registration procedure. For each motion tracking frame, a coordinate system was created for each marker triad. Rigid-body transformations were then applied to align the 3D models with the recorded motion of the marker triad. To allow comparison between participants the mandibular motion data were converted to an anatomical coordinate system, with origin at the maxillary interincisal point and aligned to the occlusal, sagittal and frontal planes, as has been done previously (Fig. 1c) (Woodford et al. 2023). Mandibular width was calculated as the distance between the left and right condylar points and mandibular length as the distance between the intercondylar point and the mandibular central incisor point (Fig. 1d).

### Bite force calculations

**Fig. 2:**
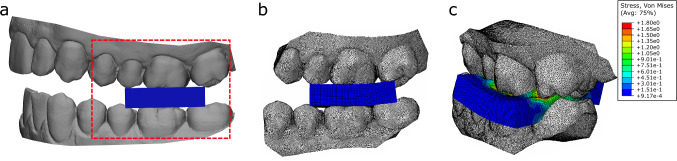
Three-dimensional model of the participants mandibular and maxillary teeth with rubber held between the first molars (**a**), finite element model of the first molars (grey) and rubber (blue) constructed from the laser scan data (**b**) and the finite element model of the first molars compressing the rubber sample in a maximum force bite (**c**). The finite element model includes only the teeth that will contact the rubber for computational efficiency, indicated by the red dashed lines

Finite element analysis was used to calculate bite force during the maximum compression biting task, following the authors previously published protocol (Woodford et al. 2021). The 3D model of each participant’s dental arches was meshed using triangular shell elements with an average edge length of 0.25 mm and imported into a commercial finite element analysis package (Abaqus 2017, Dassault Systems, Paris, France). Rubber sample geometry was created in the finite element model and aligned with the molars to match the experimental sample placement (Fig. 2a). Its volume was meshed with hexahedral elements with edge lengths of 0.40 mm in the compressed region and 1.00 mm elsewhere (Fig. 2b). The rubber elements were assigned visco-hyperelastic material properties computed through stress relaxation and compression testing (see Supplementary Material). Given the high stiffness of tooth enamel relative to rubber, the teeth were modelled as rigid bodies.

The recorded mandibular motion was used to drive finite element simulations of the mandibular and maxillary teeth compressing the rubber sample during maximum force biting (Fig. 2c). Kinematics were applied to the mandible at each timepoint by applying the resultant rotation and displacement, while the maxillary teeth were fixed in space. Tooth–rubber interaction was modelled with a general contact algorithm. To avoid tangential sliding of the rubber surface over the teeth “hard” normal and “rough” tangential contacts were employed, this was representative of the experimental conditions. To improve computational efficiency while maintaining model accuracy, mass scaling was conservatively applied to the rubber model (see Supplementary Material). Bite forces were expressed as the reaction force on the mandibular teeth with respect to the anatomic coordinate system. This finite element model was validated, demonstrating an RMS error of 1.4 N for bite forces of 160 N (Woodford et al. 2021). Each bite was normalised to 0–100% of the bite cycle, which was defined as the time the net bite force gradient first exceeded 0.3 N/s (0%) and the time at maximum net bite force (100%). The 3D bite force for each participant was averaged across the cycles. Using high-performance computing with an Intel Xeon Gold 3.10 GHz processor and 400 GB memory, dynamic simulations were executed with parallelisation over 20 cores, taking an average of 16.4 ± 10.3 h to solve.

### Musculoskeletal modelling

**Fig. 3:**
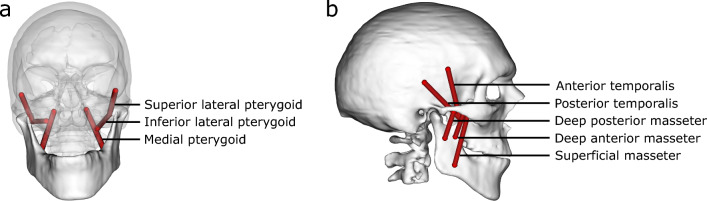
Rigid-body musculoskeletal model illustrating the pterygoid sub-regions in the frontal plane (**a**) and temporalis and masseter sub-regions in the sagittal plane of healthy controls (**b**). The superior and inferior sub-regions of the operated lateral pterygoid muscles are removed in the models of unilateral total TMJR patients

A musculoskeletal model of each participant’s jaw was constructed to estimate subject-specific muscle and joint forces at the TMJ. Open-source modelling software (Delp et al. 2007) was employed to create a generic rigid-body jaw model, with bony geometries and muscle architecture based on a previously published model (see Supplementary Material) (Ackland et al. 2017). The mandible articulated within the skull through two six degree-of-freedom TMJs. The anterior and posterior temporalis muscles, along with the superficial, deep anterior and deep posterior masseter muscles, and the medial, inferior lateral and superior lateral pterygoid muscles were modelled as Hill-type muscle–tendon actuators with muscle–tendon parameters derived from cadaveric studies (Fig. 3) (Koolstra and Eijden 2006). The generic mandible and skull anatomy, and the muscle paths were anisotropically scaled using the intercondylar distance and the distance between the mid-condyle point and the mandibular incisors of each participant. During scaling, a constant ratio of optimum fibre length and tendon slack length was maintained for each muscle.

Each participant’s time-varying bite force, calculated through finite element analysis, was applied to the mandibular first molars in the musculoskeletal model with the mandible in the maximum occlusion position. Bite force was decomposed into muscle and joint forces using an objective function that minimised the sum of squares of the muscle forces while adhering to force and moment equilibrium constraints. This objective function successfully recruits the masticatory muscles while allowing for asymmetric muscle function (May et al. 2001; Ackland et al. 2015). The superior and inferior sub-regions of the lateral pterygoid muscle were removed on the ipsilateral side for unilateral total TMJR patients, which reflected the loss of muscle function resulting from intraoperative severing of the lateral pterygoid muscle during condylectomy. All remaining muscles were modelled as intact and fully functional (see Supplementary Material).

To quantify the relative contribution of each participants working side versus non-working side muscles (Fig. 1d), an asymmetry index was computed for each muscle, defined as

1 $${\text{Assymetry index}}=\frac{{F}_{{\text{working}}} -{ F}_{{\text{non}}-{\text{working}}}}{{F}_{{\text{working}} }+ {F}_{{\text{non}}-{\text{working}}}}$$

where ${F}_{{\text{working}}}$ is the muscle force on the working side and ${F}_{{\text{non}}-{\text{working}}}$ is the muscle force on the non-working side. Positive asymmetry index values up to 1 signify that the muscles on the working side exert greater force than those on the non-working side; similarly, negative asymmetry index values up to − 1 suggest higher muscle forces on the non-working side (Ferrario and Sforza 1994).

### Data analysis

Mann–Whitney *U* tests were used to compare maximum bite force magnitude and direction, maximum muscle force, muscle asymmetry index, maximum joint force and direction and the ratio of TMJ force to bite force between unilateral total TMJR patients and controls. Wilcoxon signed-rank tests were used to compare these variables between unilateral total TMJR patients during biting on the contralateral and ipsilateral molars. Data normality was assessed using Shapiro–Wilk tests. Due to the non-normal data distribution, comparisons were made using median differences, while data dispersion was indicated by standard deviation. The criterion for statistical significance was set at *p* < 0.05. The statistical analysis was performed in Minitab version 19 (Minitab LLC, Pennsylvania, USA).

## Results

**Fig. 4:**
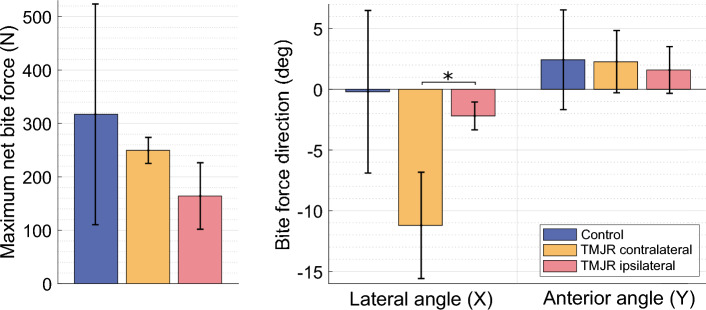
Net maximum voluntary bite force and direction for control subjects (blue) and unilateral total TMJR patients biting on their contralateral (yellow) or ipsilateral (pink) first molars. Bite force is reported as the reaction force on the mandibular teeth with respect to the mandibular anatomic coordinate system. The lateral component of bite force directed buccally is defined as positive, and that directed lingually is defined as negative. Statistically significant differences (*p* < 0.05) are identified by an asterisk

There was a non-significant trend in control subjects having a higher net maximum voluntary bite force (317.1 ± 206.6 N) than unilateral total TMJR patientswhen biting on their contralateral molars (249.6 ± 24.4 N) and their ipsilateral molars (164.2 ± 62.3 N) (Table 2). When biting on the contralateral molars, unilateral total TMJR patients had significantly higher bite force angle directed lingually than when biting on their ipsilateral molars (median difference: 7.3°, *p* = 0.048) (Fig. 4), which resulted in significantly higher lateral shear force directed lingually (median difference: 41.2 N, *p* = 0.048) (Table 2).

**Table 2:** Maximum voluntary bite force magnitude and direction for control subjects and unilateral total TMJR patients when biting on their first molars. Values are given as mean (SD). Bite force is reported as the reaction force on the mandibular teeth with respect to the anatomic coordinate system. To aid comparison between biting sides, the lateral component of bite force directed buccally is defined as positive, and that directed lingually is defined as negative. *p* values are given for comparisons between healthy controls and unilateral total TMJR patients, determined through Mann–Whitney *U* tests (${P}_{a}$) and for comparisons between unilateral total TMJR patients biting on their contralateral and ipsilateral molars, determined through Wilcoxon signed-rank tests (${P}_{b}$). *Represents significant differences

	Biting side	Maximum bite force (N)												Bite force direction (°)					
		X			Y			Z			Total			X			Y		
		Force	*P*_a_	*P*_b_	Force	*P*_a_	*P*_b_	Force	*P*_a_	*P*_b_	Force	*P*_a_	*P*_b_	Direction	*P*_a_	*P*_b_	Direction	*P*_a_	*P*_b_
Control (*n* = 8)	Both	− 9.5 (62.8)			10.0 (19.8)			− 312.8 (202.7)			317.1 (206.6)			− 0.2 (6.7)			2.4 (4.1)		
TMJR (*n* = 5)	Contralateral	− 48.8 (21.2)	0.156		9.9 (10.9)	0.897		− 243.9 (23.1)	0.699		249.6 (24.4)	0.699		− 11.2 (4.4)	0.093		2.3 (2.6)	0.897	
	Ipsilateral	− 6.0 (4.1)	0.456	0.048*	4.7 (5.6)	0.241	0.596	− 163.9 (62.1)	0.070	0.216	164.2 (62.3)	0.070	0.216	− 2.2 (1.1)	0.456	0.048*	1.6 (1.9)	0.456	0.860

**Fig. 5:**
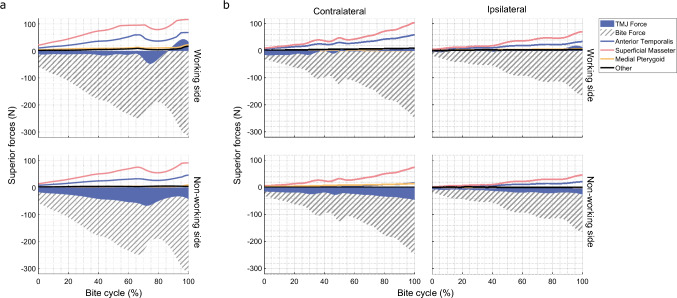
Contribution of joint force and dominant muscle forces to the superior–inferior component of bite force during a maximum force bite for control subjects (**a**) and unilateral total TMJR patients when biting on their contralateral and ipsilateral molars (**b**). Anterior temporalis (blue), superficial masseter (pink) and medial pterygoid (yellow) forces are indicated separately; contribution from remaining muscles is summed and represented as “other” (black)

**Fig. 6:**
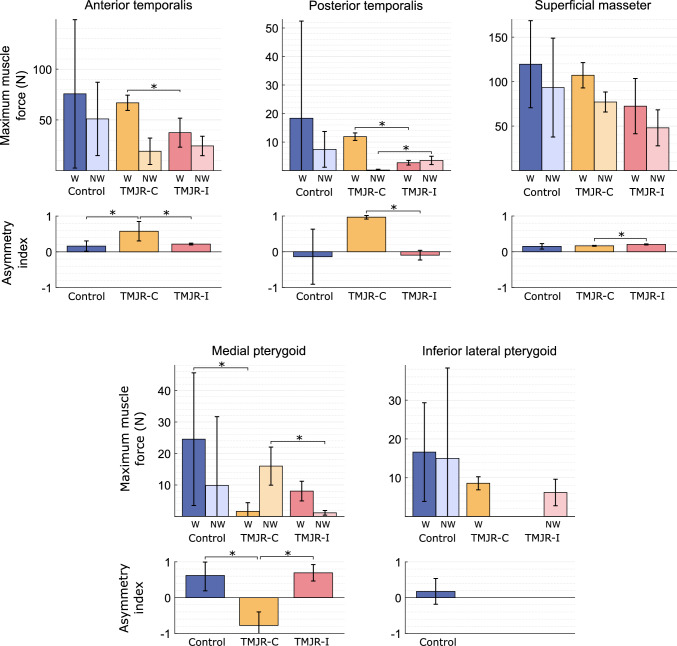
Net maximum muscle force and asymmetry index of the medial pterygoid, inferior lateral pterygoid, posterior temporalis, anterior temporalis, and superficial masseter muscles for control subjects (blue) and unilateral total TMJR patients biting on their contralateral (yellow) or ipsilateral (pink) first molars. Muscle forces are given for the working (W) and non-working (NW) sides of the jaw. Statistically significant differences (*p* < 0.05) are identified by an asterisk. TMJR-C and TMJR-I indicate unilateral total TMJR patients biting on their contralateral and ipsilateral molars, respectively

The superficial masseter, anterior temporalis and medial pterygoid muscles were the highest contributors to bite force, a finding consistent across subject groups (Fig. 5). The working side muscles exhibited higher muscle forces than the non-working side muscles, with the exception of the posterior temporalis during unilateral ipsilateral molar biting (working: 2.7 ± 0.8 N, non-working: 3.6 ± 1.5 N) and the medial pterygoid during unilateral contralateral molar biting (working: 1.6 ± 2.7 N, non-working: 16.0 ± 6.0 N) (Table 3). Unilateral total TMJR patients, when biting on their contralateral molars, had a significantly lower medial pterygoid asymmetry index than when biting on their ipsilateral molars (median difference: − 1.50, *p* = 0.048) or compared to the control subjects (median difference: − 1.34, *p* = 0.028) (Fig. 6). When biting on the contralateral molars, unilateral total TMJR patients had a significantly higher posterior temporalis asymmetry index than when biting on their ipsilateral molars (median difference: 1.10, *p* = 0.048). Unilateral total TMJR patients, when biting on the contralateral molars, had significantly higher anterior temporalis asymmetry index than when biting on their ipsilateral molars (median difference: 0.24, *p* = 0.048) and when compared to control subjects (median difference: 0.31, *p* = 0.028) (Fig. 6).

**Table 3:** Maximum muscle force magnitude for each muscle on the working and non-working side of the mandible and the asymmetry index of each muscle for each participant group. Values are given as mean (SD). *p* Values are given for comparisons between healthy controls and unilateral total TMJR patients, determined through Mann–Whitney *U* tests (${P}_{a}$) and for comparisons between unilateral total TMJR patients biting on their contralateral and ipsilateral molars, determined through Wilcoxon signed-rank tests (${P}_{b}$). *Represents significant differences

	Muscle	Control (*n* = 8)		TMJR (*n* = 5)									
				Contralateral biting				Ipsilateral biting					
		Maximum muscle force (N)	Asymmetry index	Maximum muscle force (N)	*P*_a_	Asymmetry index	*P*_a_	Maximum muscle force (N)	*P*_a_	*P*_b_	Asymmetry index	*P*_a_	*P*_b_
Working side	Anterior temporalis	75.7 (73.1)	0.16 (0.14)	66.9 (7.5)	0.366	0.58 (0.27)	0.028*	37.5 (14.3)	0.241	0.048*	0.22 (0.02)	0.594	0.048*
	Posterior temporalis	18.4 (34.0)	− 0.14 (0.77)	11.9 (1.3)	0.519	0.97 (0.05)	0.156	2.7 (0.8)	0.749	0.048*	− 0.09 (0.14)	1.000	0.048*
	Superficial masseter	119.6 (49.0)	0.15 (0.08)	107.2 (14.2)	1.000	0.16 (0.01)	0.519	72.5 (31.0)	0.110	0.377	0.21 (0.01)	0.070	0.048*
	Deep anterior masseter	5.6(6.5)	0.21 (0.17)	3.9 (0.6)	0.699	0.49 (0.07)	0.049*	2.2 (0.8)	0.070	0.112	0.27 (0.10)	0.456	0.048*
	Deep posterior masseter	4.4(6.7)	0.91 (0.07)	1.9 (0.2)	0.897	0.67 (0.05)	0.028*	1.3 (0.5)	0.110	0.216	0.82 (0.10)	0.166	0.112
	Medial pterygoid	24.5 (21.1)	0.62 (0.43)	1.6(2.7)	0.049*	− 0.78 (0.38)	0.028*	8.1 (3.1)	0.166	0.112	0.69 (0.23)	0.915	0.048*
	Inferior lateral pterygoid	16.6 (12.7)	0.18 (0.36)	8.6(1.7)	0.519	–	–	–	–	–	–	–	–
	Superior lateral pterygoid	0.2 (0.5)	0.67 (0.26)	0.0 (0.0)	0.699	–	–	–	–	–	–	–	–
Non-working side	Anterior temporalis	51.0 (36.2)		19.1 (13.0)	0.245			24.3 (9.5)	0.110	1.000			
	Posterior temporalis	7.4(6.3)		0.2 (0.3)	0.093			3.6 (1.5)	0.456	0.048*			
	Superficial masseter	93.3 (55.6)		77.1 (11.3)	0.897			48.0 (20.3)	0.070	0.112			
	Deep anterior masseter side	3.0(1.9)		1.3 (0.1)	0.093			1.4(0.7)	0.110	0.596			
	Deep posterior masseter	0.1 (0.0)		0.4(0.1)	0.028*			0.1 (0.0)	0.456	0.048*			
	Medial pterygoid	9.9 (21.8)		16.0 (6.0)	0.156			1.1 (0.7)	1.000	0.048*			
	Inferior lateral pterygoid	15.0 (23.3)		–	–			6.2 (3.4)	0.749	–			
	Superior lateral pterygoid	0.0 (0.0)		–	–			0.0 (0.0)	0.070	–

**Fig. 7:**
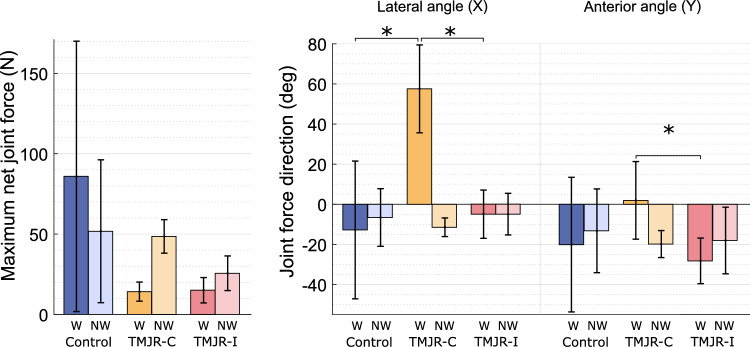
Maximum net joint force and direction for the working (W) and non-working (NW) joints of control subjects (blue) and unilateral total TMJR patients biting on their contralateral (yellow) or ipsilateral (pink) first molars. To aid comparison of the muscle and TMJ reaction loads between biting sides, the lateral component of joint force directed laterally is defined as positive, and that directed medially is defined as negative. Statistically significant differences (*p* < 0.05) are identified by an asterisk. TMJR-C and TMJR-I indicate unilateral total TMJR patients biting on their contralateral and ipsilateral molars, respectively

**Fig. 8:**
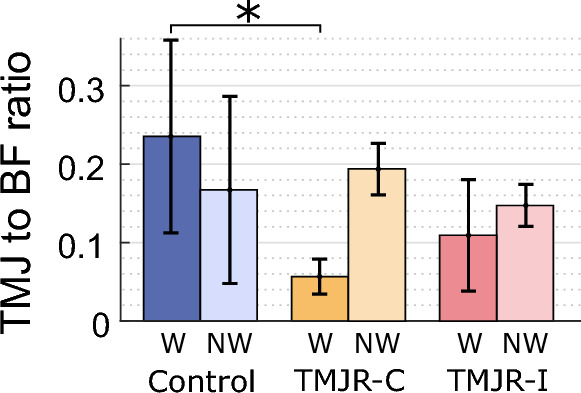
Ratio of TMJ loading to bite force for the working (W) and non-working (NW) joints of control subjects (blue) and unilateral total TMJR patients biting on their contralateral (yellow) or ipsilateral (pink) first molars. Statistically significant differences (*p* < 0.05) are identified by an asterisk. TMJR-C and TMJR-I indicate unilateral total TMJR patients biting on their contralateral and ipsilateral molars, respectively

Control subjects had similar mean net joint forces on both TMJs (working: 86.0 ± 84.1 N, non-working: 51.8 ± 44.4 N). There was a non-significant trend in unilateral total TMJR patients having a higher net joint forces on their non-working condyle than their working condyle, both for biting on the contralateral molars (working: 14.2 ± 5.9 N, non-working: 48.5 ± 10.4 N) and the ipsilateral molars (working: 15.1 ± 7.9 N, non-working: 25.6 ± 10.8 N) (Fig. 7). All subject groups experienced compressive mean joint forces in the superior/inferior direction on the non-working condyle (control: − 43.0 ± 43.1 N, unilateral contralateral: − 44.3 ± 7.3 N, unilateral ipsilateral: − 24.9 ± 10.9 N) and tensile forces on the working condyle (control: 33.7 ± 116.3 N, unilateral contralateral: 6.0 ± 1.8 N, unilateral ipsilateral: 13.5 ± 8.1 N) (Table 4). When biting on the contralateral molars, the working joint of unilateral total TMJR patients had a significantly higher lateral force direction compared to that when biting on their ipsilateral molars (median difference: 60.1°, *p* = 0.048) or that of control subjects (median difference: 63.6°, *p* = 0.028). Unilateral total TMJR patients, when biting on the contralateral molars, had a significantly lower ratio of TMJ force to bite force on the working condyle than that of control subjects (median difference: 0.17, *p* = 0.049) (Fig. 8).

**Table 4:** TMJ reaction force magnitude, direction and ratio of TMJ force to bite force for the working and non-working condyles of control subjects and unilateral total TMJR patients at the time of maximum voluntary bite force. Values are given as mean (SD). To aid comparison of the muscle and TMJ reaction loads between biting sides, the lateral component of joint force directed laterally is defined as positive, and that directed medially is defined as negative. Values are given as mean (SD). *p* values are given for comparisons between healthy controls and unilateral total TMJR patients, determined through Mann–Whitney *U* tests (${P}_{a}$) and for comparisons between unilateral total TMJR patients biting on their contralateral and ipsilateral molars, determined through Wilcoxon signed-rank tests ${P}_{b}$). * represents significant differences

	Biting side	TMJ	TMJ force (N)												TMJ force direction (°)						TMJ to BF ratio		
			X			Y			Z			Total			X			Y					
			Force	*P*_a_	*P*_b_	Force	*P*_a_	*P*_b_	Force	*P*_a_	*P*_b_	Force	*P*_a_	*P*_b_	Direction	*P*_a_	*P*_b_	Direction	*P*_a_	*P*_b_	Ratio	*P*_a_	*P*_b_
Control (*n* = 8)	Both	Working	2.5 (20.9)			3.8 (19.0)			33.7 (116.3)			86.0 (84.1)			− 12.8 (34.3)			− 20.1 (33.6)			0.24 (0.12)		
		Non-working	− 5.0 (13.0)			− 10.7 (24.4)			− 43.0 (43.1)			51.8 (44.4)			− 6.6 (14.4)			− 13.2 (20.9)			0.17 (0.12)		
TMJR (*n* = 5)	Contralateral	Working	12.1 (7.6)	0.156		− 0.1 (1.7)	0.915		6.0 (1.8)	0.519		14.2 (5.9)	0.070		57.5 (21.9)	0.028*		1.9 (19.3)	0.519		0.06 (0.02)	0.049*	
		Non-working	− 9.4 (5.1)	0.156		− 16.7 (8.4)	0.156		− 44.3 (7.3)	0.245		48.5 (10.4)	0.699		− 11.5 (4.6)	0.915		− 19.9 (6.7)	0.245		0.19 (0.03)	0.156	
	Ipsilateral	Working	− 0.4 (1.7)	0.456	0.048*	− 5.6 (1.4)	0.749	0.048*	13.5 (8.1)	0.594	0.216	15.1 (7.9)	0.156	0.860	− 4.9 (12.0)	0.915	0.048*	− 28.2 (11.4)	0.594	0.048*	0.11 (0.07)	0.070	0.216
		Non-working	0.0 (0.8)	0.915	0.048*	− 4.4 (2.0)	0.337	0.048*	− 24.9 (10.9	0.749	0.112	25.6 (10.8)	0.594	0.112	− 4.9 (10.4)	0.915	0.377	− 18.1 (16.6)	0.456	0.377	0.15 (0.03)	0.749	0.112

## Discussion

Clinical research shows that TMJR surgery increases maximum interincisal opening distance by 9 mm and reduces joint pain and dietary restrictions by 74% and 77%, respectively (Dimitroulis et al. 2018). However, 3D bite force, muscle force and joint loads in TMJR patients, which are key determinates of muscle and joint function post-operatively, remain poorly understood. The objective of this study was to use subject-specific 3D bite force measurements to evaluate the magnitude and direction of joint loading in TMJR patients and healthy controls. There was a non-significant trend that unilateral total TMJR patients had lower net joint force than healthy controls, particularly on the contralateral joint. Unilateral total TMJR patients, when biting with their contralateral molars, had TMJ loading on the working condyle directed significantly more laterally than healthy controls and significantly lower ratios of TMJ force to bite force than healthy controls, thus supporting the study hypothesis.

Comparable net maximum voluntary bite force was observed between control subjects and unilateral total TMJR patients. Control subjects had a net maximum voluntary bite force of 317.1 ± 206.6 N, which is consistent with a previously reported unidirectional maximum voluntary bite force range for healthy females of 269.6 to 424.9 N (Calderon et al. 2006; Alam and Alfawzan 2020). There were large variations in maximum voluntary bite force observed in the control subjects. This finding reflects the variability of normal force documented in the literature, believed to be a result of variations in muscle strength, periodontal support, motor control and general physical health (Ikebe et al. 2005; Jansen van Vuuren et al. 2020). Uniaxial maximum voluntary bite force in TMJR patients, using their contralateral molars, has been reported to be 194.5 N (Linsen et al. 2021a), which is lower than the net maximum voluntary bite force measured in the present study (249.6 N); however, this study did not record shear force at the occlusal surface during contralateral biting, which may underestimate the resultant occlusal load, although Linsen et al. studied a mixed-gender sample (15 M, 24 F) which may increase the group-average occlusal loading. From a functional standpoint, we showed that all participants had mean maximum voluntary bite force sufficient to chew raw carrot (118 N) or cooked meat (124 N) (Poli et al. 2021), suggesting that unilateral total TMJR surgery does not result in a functional limitation to biting performance. This is supported by clinical studies finding high levels of satisfaction and reduction in dietary restrictions following total TMJR surgery with the ArthroJaw TMJR system (Dimitroulis et al. 2018).

Large lateral shear forces of 49 ± 21 N were measured in unilateral total TMJR patients when biting on the contralateral molars. To our knowledge, this is the first study to measure 3D maximum voluntary bite force following total TMJR surgery and therefore the first quantification of lateral shear forces in this cohort. This finding suggests that these patients rely on a grinding motion to breakdown food when biting on the contralateral molars. From a clinical standpoint, these shear forces ought to be considered when planning dental treatments for unilateral total TMJR patients, as comparable shear forces have been shown to propagate cracks in dental enamel (~ 100 N) (Chai et al. 2009), lead to failure of restored teeth (92 N) (Wandscher et al. 2014) and dental implants (106 N) (Forero et al. 2019).

The present study showed that in unilateral total TMJR patients, both the posterior and anterior temporalis muscles have lower forces on the operated side, leading to larger muscle asymmetry than healthy controls. This is consistent with studies finding large temporalis asymmetry following TMD surgery (Raustia et al. 1997). Contraction of the temporalis muscle elevates the mandible and produces vertical bite force when chewing; thus, this post-surgical weakness may limit the maximum voluntary bite force of TMJR patients, particularly when biting on the ipsilateral side. Unilateral total TMJR patients had high medial pterygoid forces on the operated side, regardless of biting side. This may be a compensation for the loss of the inferior lateral pterygoid function, since animal studies have shown that the absence of an individual masticatory muscle may be compensated for by ipsilateral synergistic muscle function (Rafferty et al. 2012). Despite this compensation, the loss of the lateral pterygoid muscle has been associated with decreased anterior motion of prosthetic condyles, leading to reduced jaw movement capacity (Wojczyńska et al. 2021; Woodford et al. 2023). This study found low force generation in the deep masseter muscles, which is consistent with another musculoskeletal model of the masticatory system (Ackland et al. 2017); however, fine wire EMG studies predict larger activation of the deep masseter, and this may represent a limitation in the muscle models employed, particularly their associated cost functions and constraints (Van Eijden et al. 1993).

During TMJR surgery, the insertion of the masseter muscle, the pterygomasseteric sling, is partially stripped from the mandible. After fixation of the ramal component, the pterygomasseteric sling can be reattached via sutures, which would be expected to decrease masseter forces on the operated side (Linsen et al. 2020). However, this study found similar levels of masseter asymmetry between controls and unilateral total TMJR patients, indicating healthy post-surgical masseter function in the patients studied. All TMJR patients in this study were fitted with the ArthroJaw customised TMJR system which has a titanium (Ti–6Al–4V) condyle-ramus unit, designed to maximise exposure of mandibular bone, facilitating reattachment of the masseter muscle (Ackland et al. 2017; Dimitroulis et al. 2018). Additionally, studies of biomaterials created for orthopaedic applications have shown high proliferation and adhesion of osteoblasts and fibroblasts on titanium implants (Markhoff et al. 2017), suggesting that the masseter may ultimately reattach to the surface of the titanium implant (Singh et al. 2022). This adhesion may lead to higher post-surgical masseter forces in patients with custom titanium alloy prosthetics compared to those with a stock cobalt chromium alloy prosthetics (Linsen et al. 2020).

Muscle and joint function in unilateral TMJR patients exhibit similarities with those affected by unilateral posterior crossbite (UPC), including mandibular asymmetry (Bell and Kiebach 2014), reverse-sequence chewing patterns (Throckmorton et al. 2001), muscle asymmetries (Piancino et al. 2016) and reduced ipsilateral bite force capacity (Sonneson et al. 2001; Sonneson and Bakke 2007). If left untreated, asymmetric jaw function in UPC patients may lead to TMJ dysfunction (Thilander et al. 2002; Thilander and Bjerklin 2012) and abnormal bone remodelling at the mandibular condyle (Bell and Kieback 2014). Orthodontic treatment to achieve mandibular symmetry has been successful in restoring normal jaw function in UPC patients (Throckmorton et al. 2001; Piancino et al. 2016), suggesting that surgical approaches targeting muscle and joint symmetry at the mandible may also be beneficial for TMJR patients.

Unilateral total TMJR surgery is associated with degradation of the native contralateral TMJ. It changes the morphology of the native joint (Kim et al. 2021) and leads to subsequent bilateral total TMJR surgery in up to 40% of cases (Franco et al. 1997). Previous investigations have speculated that this is due to altered loading patterns resulting from hypermobility of the native joint (Leiggener et al. 2012) or joint overloading (Perez et al. 2016). However, this study found that for unilateral total TMJR patients, the native side of their jaw is more efficient at transmitting muscle force to bite force than the prosthetic side, as evidenced by the low joint loads and ratio of TMJ force to bite force for the native contralateral joint. These findings follow a similar trend to mathematical models of TMJ loading for unilateral total TMJR patients (van Loon et al. 1998) and suggest that joint disease on the native contralateral TMJ may not result from joint overloading during biting. Alternatively, as unilateral biting is the dominant loading event to the masticatory system, this degradation may be due to joint underloading, which has been shown to lead to degradation in the knee (Vanwanseele et al. 2002; Wellsandt et al. 2016) and hip (Loureiro et al. 2018) joints.

The results reported in this study are not direct biomechanical measurements, but are indirect estimates calculated using computational models. Direct in vivo measurements of 3D bite force and TMJ loading are currently not possible, and this has constrained our understanding of maxillofacial biomechanics in both healthy and pathological populations. Our technique of combining experimental measurements and computational modelling, referred to as in-silico modelling, has been applied widely in the field of orthopaedic biomechanics and is shown to provide valid measures of joint loading in the hip, knee, ankle and shoulder joints (Innocenti et al. 2022). Established, validated musculoskeletal models of the TMJ are limited due to the sheer complexity of the masticatory system, which includes the force-generating capacity of the musculature, neuromuscular control of jaw function and the interaction between hard and soft tissues, as well as their variability. However, the present study demonstrates the potential for computational modelling to expand the understanding of TMJ biomechanics where in vivo quantification is not possible.

There are a number of limitations of this study. The small sample size may have underpowered our capacity to detect significant differences in maximum voluntary bite force considering the large variation in maximum voluntary bite force for each subject group. However, our sample size was sufficient to identify significant differences in bite force direction, muscle forces and asymmetries, TMJ loading direction and ratio of TMJ force to bite force. Additionally, this study involved only female participants, which ought to be considered when interpreting these results. This was chosen to reflect the gender imbalance among TMJR patients of whom 86.1% are female (Granquist et al. 2020). This is believed to stem from differing behavioural, psychosocial and hormonal factors (Roda et al. 2007). Further research is required to assess jaw biomechanics in the male population. Our use of dental plates for motion tracking of the jaw may introduce kinematics measurement errors through movement or vibration of the markers, mismatch between the alignment of the rubber sample with respect to the teeth between the model and experiments, and assumptions in the modelling of rubber–clasp interactions (Supplementary Material). Furthermore, for unilateral total TMJR patients, all muscles were modelled as intact with full force capacity, with the exception of the superior and inferior sub-regions of the lateral pterygoid muscle which were modelled as completely severed (Bhargava et al. 2019; Pinheiro 2021). Sensitivity analysis shows that this introduces minimal error in calculations of muscle and joint forces (see Supplementary Material). However, future studies ought to employ MRI or EMG to quantify the status of the pterygoid, masseter and temporalis muscles on a subject-specific basis. Finally, anisotropically scaled generic rigid-body musculoskeletal models were used instead of fully subject-specific models, and this may introduce offsets in joint centre location and muscle attachment points. While previous studies have shown that small differences in TMJ location have only minor influence on muscle and joint loading (van Loon et al. 1998), future studies should create fully subject-specific models using MRI and CT data to determine precise muscle insertion and joint centre locations to increase the accuracy of these results.

This study found that unilateral total TMJR patients tended to have lower maximum voluntary bite force than that of healthy controls, particularly when biting on their ipsilateral molars. This may be linked to an asymmetric force distribution between the contralateral and ipsilateral temporalis muscles. Additionally, unilateral total TMJR patients had lower net TMJ loading than controls, particularly on the contralateral joint. This under-loading may be a mechanism associated with degenerative changes that have been observed in the contralateral TMJ following unilateral total TMJR surgery. The results of this study may facilitate the biomechanical design and evaluation of maxillofacial implants, prosthetic devices and dental restorations, to improve their long-term performance. Furthermore, these results may influence physiotherapy prescription following unilateral total TMJR surgery, for example, to prioritise strengthening of the operated elevator muscles and increasing the loading of the contralateral joint.

### Supplementary Information

Below is the link to the electronic supplementary material.

Supplementary file1 (DOCX 1735 KB)
