# Supplementary material for: Muscle and joint mechanics during maximum force biting following total temporomandibular joint replacement surgery
Source: Biomech Model Mechanobiol. 2024 Mar 19;23(3):809–23. doi: 10.1007/s10237-023-01807-1 (PMC11101553; doi:10.1007/s10237-023-01807-1)
Supplement: Supplementary file 1 — Supplementary file1 (DOCX 1735 KB) [file 10237_2023_1807_MOESM1_ESM.docx]

Supplementary Material

**BITE FORCE CALCULATION**

*Motion tracking plate validation*

The motion tracking error was evaluated in one male participant (age: 41 years) with healthy dentition and no history of dental pain or mandibular disorders. Subject-specific motion tracking plates were constructed for this subject as per the study protocol (see Materials and Methods). The participant had a lip and cheek retractor inserted to expose the teeth and was instructed to insert the two motion tracking plates. Following this, six 4 mm hemispherical retroreflective markers were fixed to the incisors and premolars using dental adhesive, three on the maxillary teeth and three on the mandibular teeth (Figure S1). Three 4mm hemispherical retroreflective markers were attached to each plate (Fig S1) to allow simultaneous motion tracking of the tooth mounted and plate mounted markers. The participant was instructed to chew on a rubber sample whilst the positions of the 12 retroreflective markers were recorded using an optoelectronic tracking system sampling at 100Hz and filtered using a low-pass filter with a cut-off frequency of 10Hz. Motion tracking using the teeth mounted markers represented the “gold standard” measurement of jaw motion. For each motion capture frame, the distance between the centrode of the tooth-mounted markers and the centroid of the plate-mounted markers was calculated. The resulting standard deviation of this distance (0.13 mm) was used as a measure of motion measurement accuracy of the tracking plates in the present study.


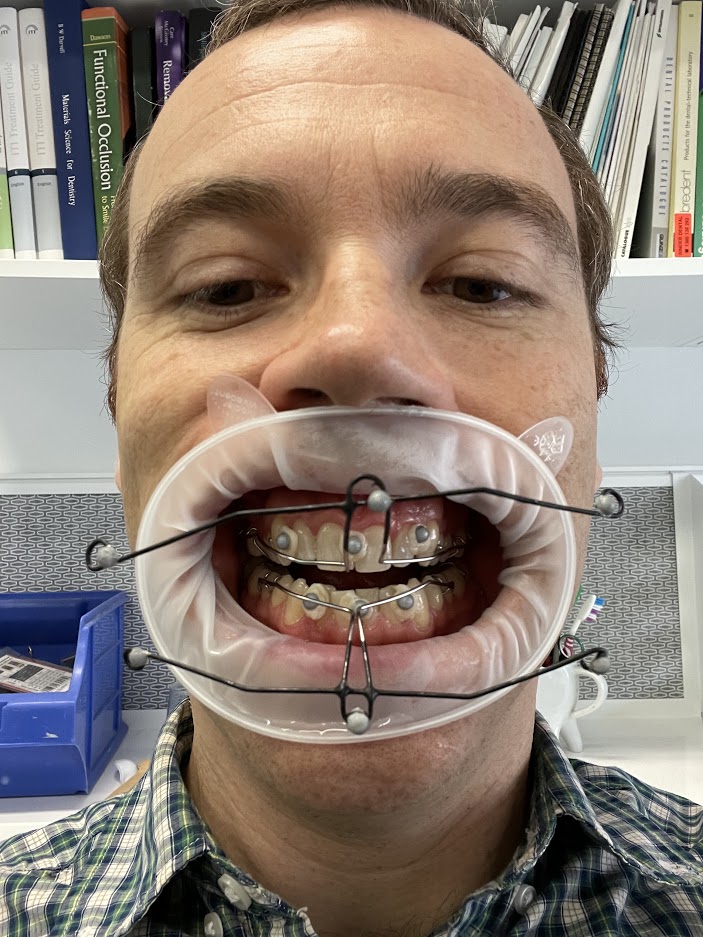


Figure S1: Subject wearing motion tracking plates and 4mm hemispherical retroreflective markers attached to the mandibular and maxillary incisors and premolars.

*Material characterisation*

The rubber material was modelled as a visco-hyperelastic material with a near incompressible material response. The material properties of the rubber sample were derived experimentally following the authors previously published protocol. The hyperelastic response was modelled using the phenomenological Ogden model, with the strain energy density function defined as:

$W(\lambda_{1},\lambda_{2},\lambda_{3})= \sum_{i=1}^{N} \frac{\mu_{i}}{\alpha_{i}}(\lambda_{1}^{\alpha_{i}}+\lambda_{2}^{\alpha_{i}}+\lambda_{3}^{\alpha_{i}}-3)$ (1)

Where N = 1, $\lambda_{i}$ are the principle stretch ratios and $\mu_{i}$ and $\alpha_{i}$ are the phenomenological material constants (4.60 × ${10}^{5}$ and 2.09 Pa, respectively) obtained using least squares optimisation on experimentally measured stress-strain curves ($R^{2}$ = 0.9985) (Figure S2a). The viscoelastic material response was modelled as a second order Prony series, with relaxation modulus given by:

$g_{R}\left( t \right)=1- \sum_{i=1}^{N} g_{i}(1-\exp\left( \frac{-t}{\tau_{i}} \right))$ (2)


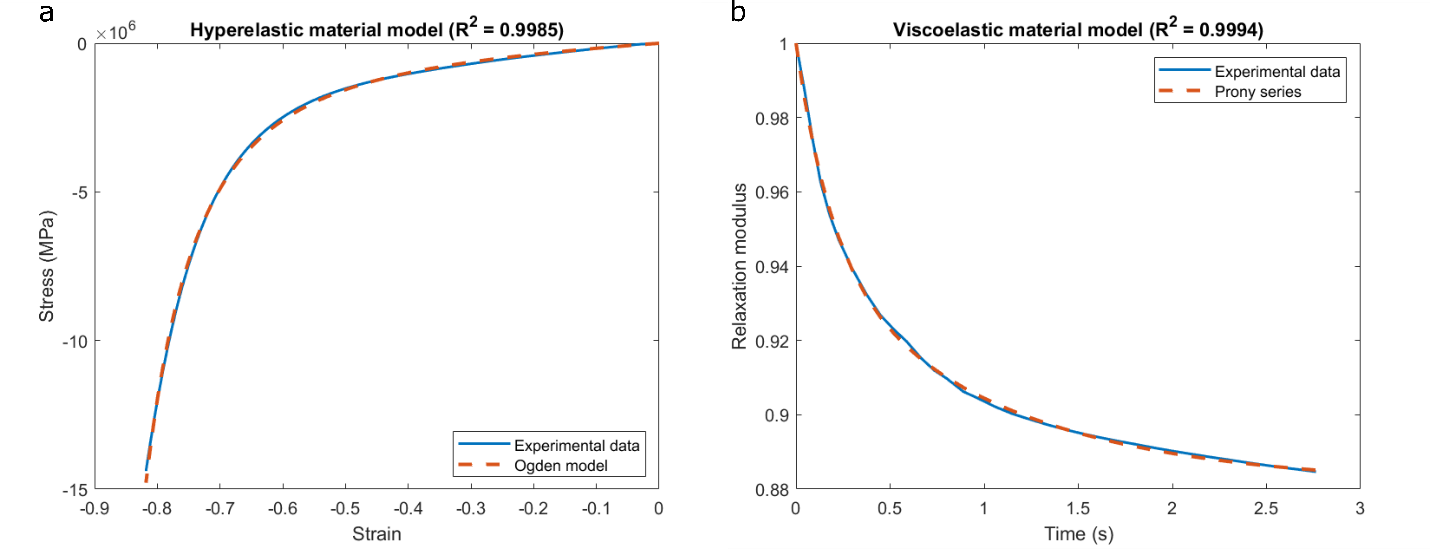
Where N = 2, $t$ is time, and $g_{i}$ and $\tau_{i}$ are the phenomenological material constants (first order: $6.30 \times{10}^{-2}$ and 0.99 s, second order: $5.58 \times{10}^{-2}$ and 0.19 s, respectively) obtained using least squares optimisation on experimentally measured stress-relaxation curves ($R^{2}= 0.9994$) (Figure S2b).

Figure S2: Material model fit to experimental data. (a) Hyperelastic material response, Ogden model (N=1) fit to average experimentally measured stress-strain data. (b) Viscoelastic material response, Prony series (N=2) fit to average experimentally measured stress-relaxation data.

*Mass scaling*

Mass scaling was applied to the simulations to reduce the computation time. To assess whether this addition still led to a meaningful solution the maximum ratio of kinetic energy to internal energy was calculated as a percentage for all simulations to ensure that inertial forces remained insignificant throughout the simulations. The mean ratio of kinetic energy to internal energy is $7.79\times{10}^{-5}$ ± $1.60{\times10}^{-4}$ %. All values remained below the accepted 2%.

*Wire interference*

Finite element simulations contained only the rigid geometry of the teeth and the deformable geometry of the rubber. The wire clasps of the mouthpieces were not included in the model. To assess the effect of this simplification, one model was created with 0.7 mm wires added around the first molars, and between the mandibular premolars (Figure S3b), to represent the mouthpiece attachment points (Figure S3a).


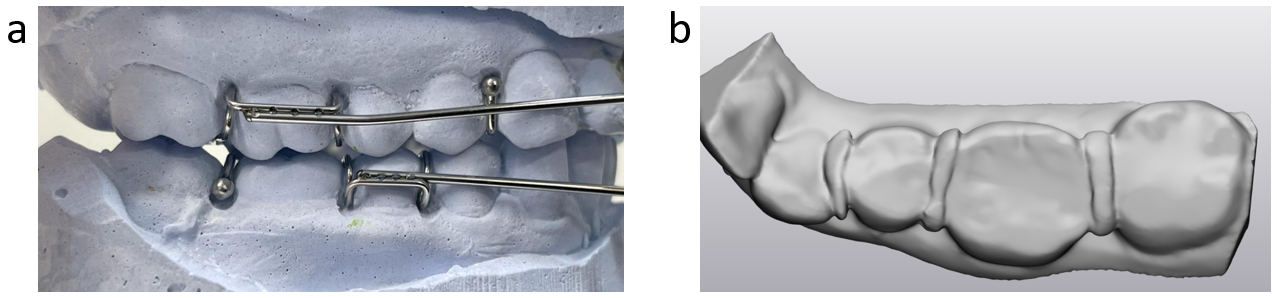


Figure S3: Dental stone casts with mouthpieces attached showing maximum occlusion position (a), model of mandibular teeth with 0.7 mm wires between the teeth (b).

Modelling the wires increased the net bite force by 36.5 N, which corresponded to an error of 11.8 % (Figure S4).


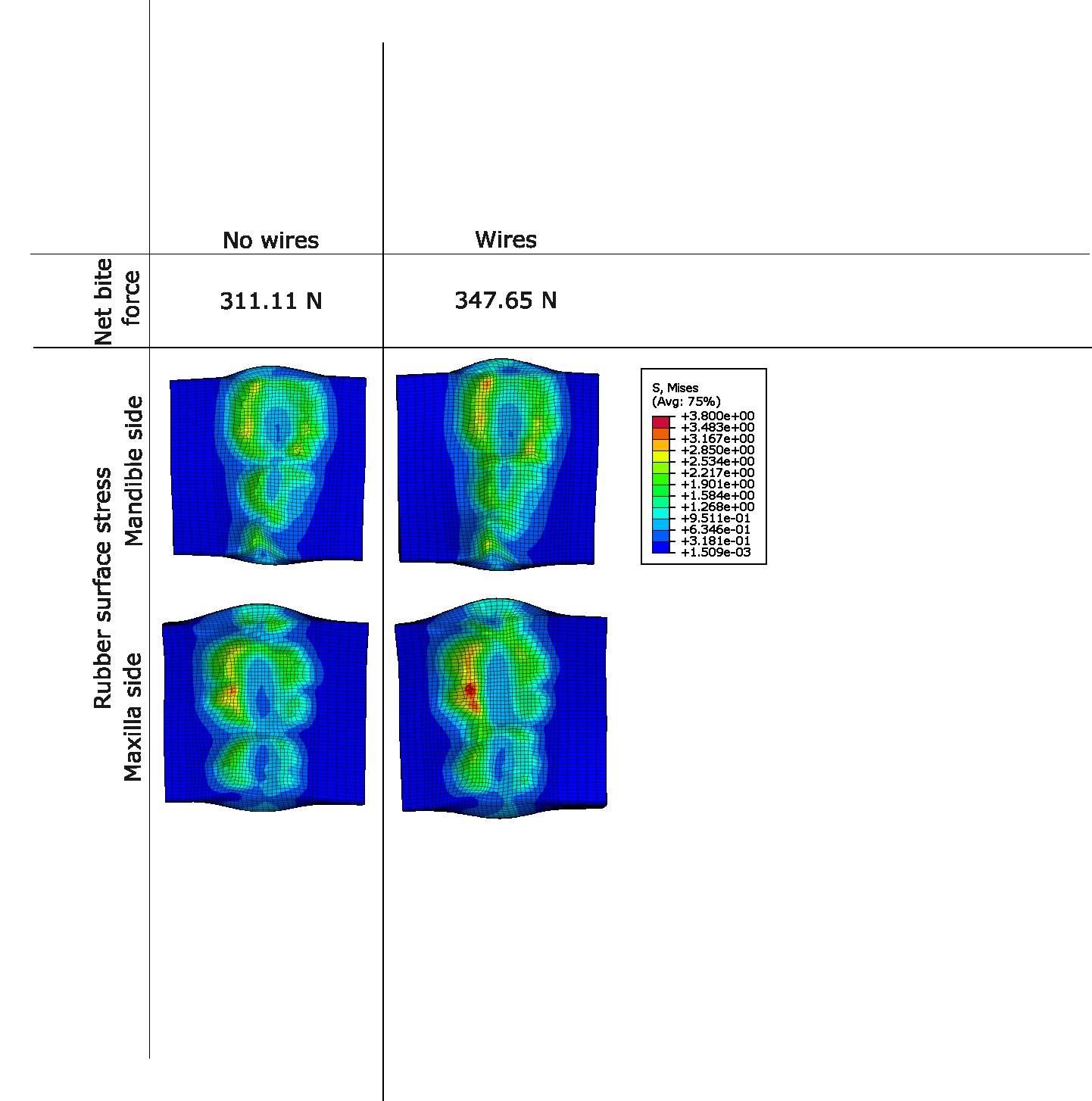


Figure S4: Results of sensitivity analysis studying the effects of the mouthpiece wires on bite force results.

**MUSCULOSKELETAL MODELLING**

Each participant’s musculoskeletal model was based on a previously published generic, unscaled rigid body model with musculotendon properties defined in Table S1 (Ackland et al. 2017). For each participant the muscle attachment points were anisotropically scaled based on their intercondylar distance and the distance between the mid-condyle point and the mandibular incisors. For each muscle a constant ratio of optimum fibre length and tendon slack length was maintained during scaling.

The effect of modelling the masseter and temporalis muscles with full capacity was investigated through a sensitivity analysis. For one total unilateral TMJR patient musculoskeletal models were setup with the maximum isometric forces of the ipsilateral anterior temporalis, posterior temporalis, superficial masseter, deep anterior masseter and deep posterior masseter muscles modelled as 50%, 60%, 70%, 80%, 90% and 100% of their full capacity. The ipsilateral inferior lateral pterygoid and superior lateral pterygoid remained removed from the model, and the applied bite force remained the same. As the ipsilateral masseter and temporalis strength is decreased there is higher influence from the contralateral superficial masseter and the ipsilateral medial pterygoid muscles (Figure S5). This resulted in higher compressive and tensile TMJ loading during ipsilateral molar biting for weakened masseter and temporalis muscles (Figure S6). There is no available literature directly compares EMG activity of the temporalis and masseter muscles of TMJR patients to healthy controls. However, studies comparing contralateral to ipsilateral muscle function of unilateral total TMJR patients found operated to non-operated muscle force ratios of 0.97 and 0.89 for the masseter and temporalis, respectively, 12 months after surgery (Raustia et al. 1997). Therefore, it is reasonable to assume that the temporalis and masseter muscles of the unilateral total TMJ replacement patients in this study were operating between 90% to 100% of their full capacity.

Table S1:Musculotendon properties of the generic, unscaled rigid body musculoskeletal model.


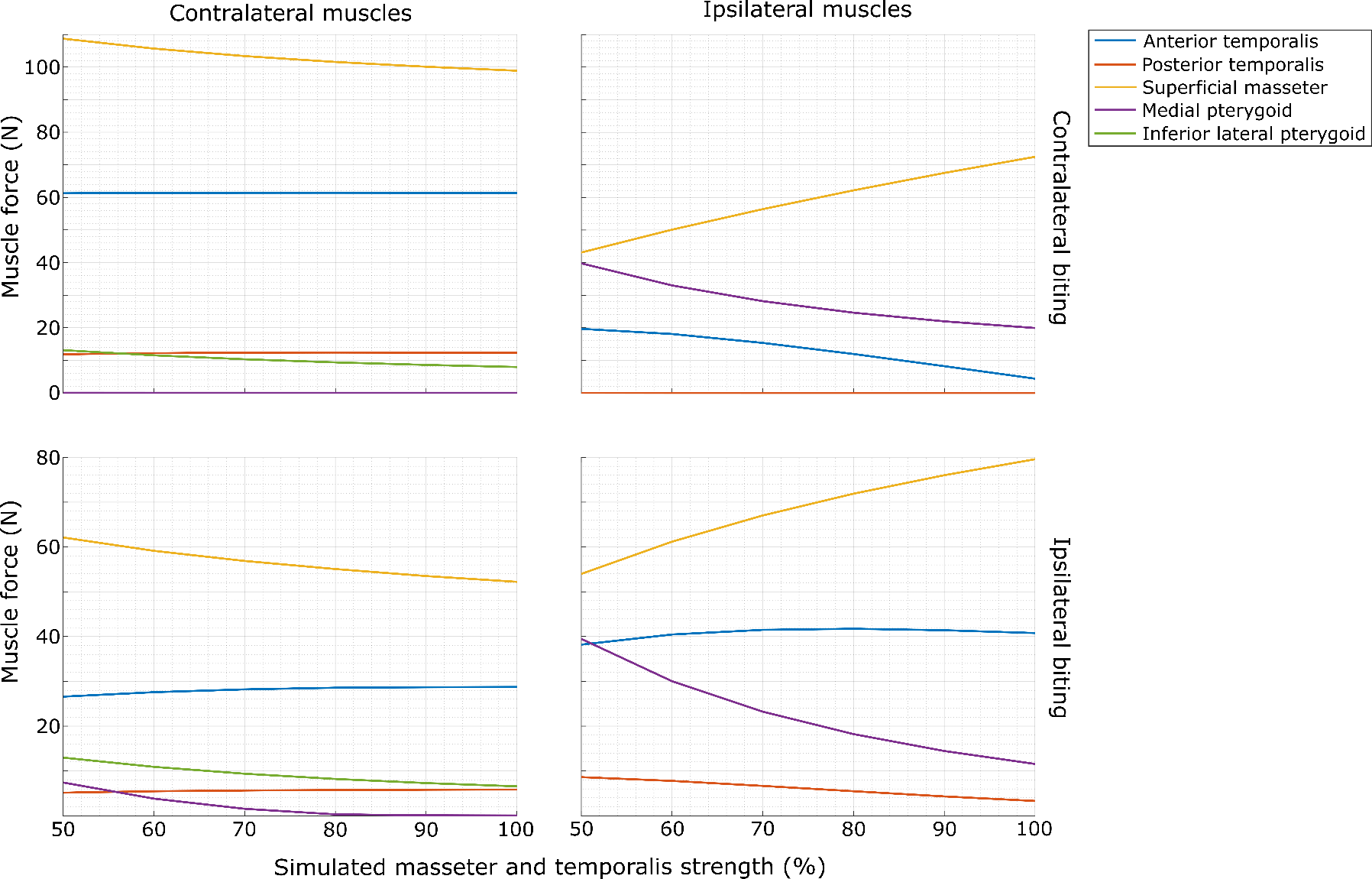


Figure S5: Resultant muscle forces for the anterior temporalis, posterior temporalis, superficial masseter, medial pterygoid and inferior lateral pterygoid muscles when the maximum isometric force of the temporalis and masseter sub-regions are modelled as 50%, 60%, 70%, 80%, 90% and 100% of their full capacity.


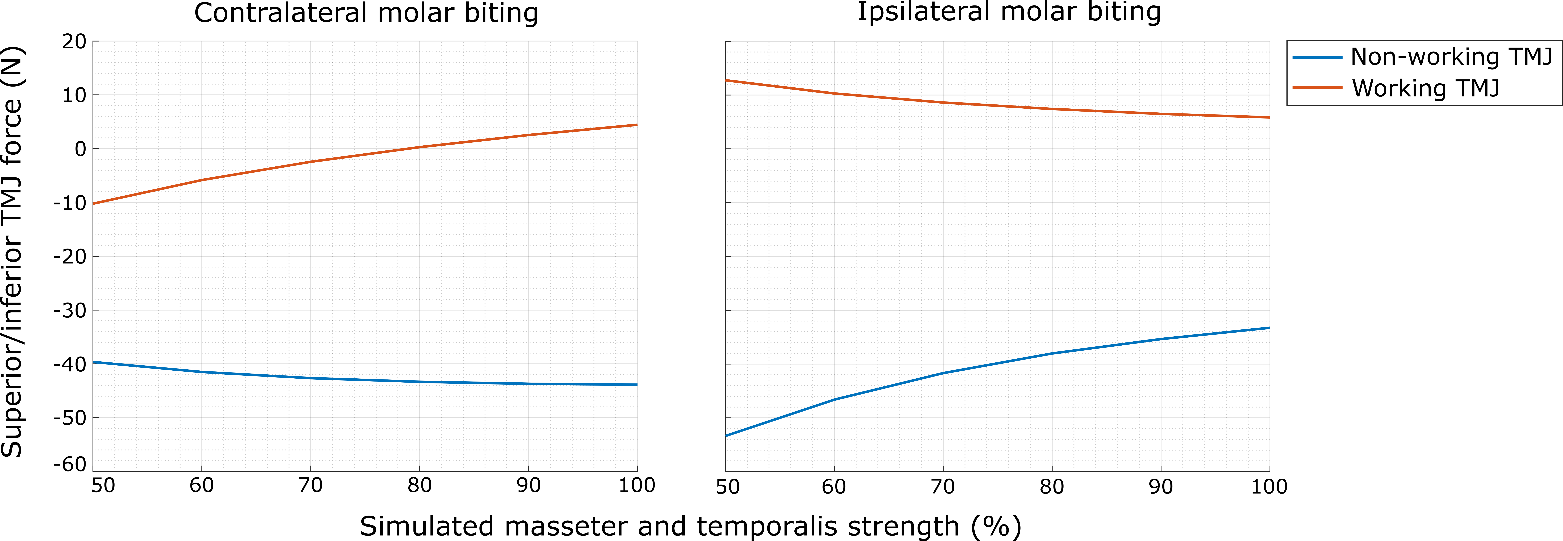


Figure S6: TMJ loading when the maximum isometric force of the temporalis and masseter sub-regions are modelled as 50%, 60%, 70%, 80%, 90% and 100% of their full capacity.

**REFERENCES**

Ackland DC, Robinson D, Redhead M, et al (2017) A personalized 3D-printed prosthetic joint replacement for the human temporomandibular joint: From implant design to implantation. J Mech Behav Biomed Mater 69:404–411

Raustia AM, Oikarinen KS, Pernu H (1997) Changes in electrical activity of masseter and temporal muscles after temporomandibular joint surgery: A one-year follow up. Int J Oral Maxillofac Surg 26:253–257
